# Privacy risk perception and privacy disclosure behavior among college students: the mediating role of online social anxiety and the moderating role of privacy cynicism

**DOI:** 10.3389/fpsyg.2026.1891374

**Published:** 2026-08-06

**Authors:** Shuyan Huang, Lizhuang Yuan, Yuxuan Yang, Jing Li, Yitan Chen, Yingjie Liu, Shuhao Zhang, Peng Liu

**Affiliations:** 1 School of Psychology and Mental Health, North China University of Science and Technology, Tangshan, China; 2 Hebei Key Laboratory of Mental Health and Brain Science, North China University of Science and Technology, Tangshan, China; 3 Department of Psychology, Hebei Normal University, Shijiazhuang, China; 4 The First Hospital of Hebei Medical University, Shijiazhuang, China; 5 School of Life Science, North China University of Science and Technology, Tangshan, China

**Keywords:** college students, online social anxiety, privacy cynicism, privacy disclosure behavior, privacy risk perception

## Abstract

**Background:**

Social media is a key platform for college students’ daily communication. However, frequent online fraud incidents have raised widespread concern. Interestingly, many users’ high-risk perception does not prevent them from continuing to disclose personal information. To further explore the privacy paradox among college students on current social networks, this study examines the relationship between college students’ privacy risk perception and privacy disclosure behavior, as well as the mechanisms of online social anxiety and privacy cynicism.

**Methods:**

A survey was conducted among 1, 276 college students using the Perceived Privacy Risk Measurement Scale, Privacy Disclosure Behavior Scale, Social Anxiety Scale for Social Media Users, and Privacy Cynicism Scale.

**Results:**

Privacy risk perception is significantly and positively associated with college students’ privacy disclosure behavior. Online social anxiety partially mediated the relationship between privacy risk perception and privacy disclosure behavior. Privacy cynicism significantly moderated the effects of privacy risk perception on online social anxiety and privacy disclosure behavior.

**Conclusion:**

These findings suggest the interaction between emotion and cognitive attitude in college students’ privacy decision-making, providing a new perspective for cybersecurity education.

## Introduction

Currently, telecommunications and cyber fraud incidents occur frequently. In China, college students have become a high-risk group vulnerable to such fraud (Tan et al., 2025). Relevant reports indicate that students born in the 2,000 s account for 18.2% of those who have fallen victim to such scams, and this group has seen the largest increase (Sun et al., 2024). Research suggests that breaches of college students’ personal information is a major cause of such fraud (Shen, 2017). As natives of social networks, college students all too often experience personal privacy breaches, resulting from inappropriate use and a lack of self-protection awareness (Zhang and Li, 2019). Privacy disclosure behavior refers to an individual’s intentional act of revealing their private or sensitive information to the external environment or others (Kolotylo-Kulkarni et al., 2021). Multiple studies have pointed out that experiences of telecommunications and cyber fraud can cause a series of negative effects on college students, such as financial burdens, negative emotional experiences, self-destructive tendencies, and even a reversal of roles leading to criminal behavior (Sun and Zhao, 2024; Li, 2007). Therefore, investigating the influencing factors and mechanisms of privacy disclosure behavior on social networks is of great practical significance for preventing college students from becoming victims of telecommunications and cyber fraud and for protecting their financial, physical, and mental well-being.

### Privacy risk perception and privacy disclosure behavior

Privacy risk perception refers to an individual’s subjective assessment of the potential negative consequences that may arise from disclosing information, including misuse of information, identity theft, and damage to one’s social reputation (Li Y. Y. et al., 2023). According to Protection Motivation Theory (PMT), risk perception, as an individual’s cognitive assessment of threats, is a core antecedent variable driving privacy protection behavior and a key cognitive factor in predicting privacy disclosure behavior (Rogers, 1975). Empirical research has showed that privacy risk perception negatively predicts privacy disclosure behavior (Zhou and Liu, 2023). When individuals have a higher perception of the risk that their personal information may be leaked or misused, they tend to reduce their disclosure of personal information.

However, in the context of social media activities, researchers have obtained different observations: although users are aware of the reality and risks of privacy breaches on social networks, this has not significantly altered the amount of personal information they disclose in their profiles (Pu and Fei, 2023). This contradiction between privacy concerns and privacy disclosure behavior is referred to as the privacy paradox (Dienlin et al., 2023). Understanding this paradox is facilitated by Privacy Calculus Theory (PCT), which provides a useful explanatory framework. The theory suggests that individuals’ privacy decisions are not based solely on their level of perceived risk, rather, they involve a contextual trade-off between expected benefits and potential risks (Culnan and Armstrong, 1999). Taddicken (2014), in a study of German internet users, systematically examined the extent and channels of information disclosure and reported no significant direct association between privacy concerns and actual disclosure behavior. Chinese scholars have also observed that, while risk perception in the context of WeChat use heightens attention to privacy issues, such awareness does not directly translate into reduced disclosure willingness or behavior (Li J. H. et al., 2023). This may be because college students are at a critical stage in establishing self-identity and expanding their social networks. Even though they express concern about potential risks to personal information security, they do not alter their self-disclosure habits and behaviors in order to seek social acceptance, maintain social connections, or access services (Pu and Fei, 2023).

The inconsistent findings outlined above suggest that the direction of the relationship between privacy risk perception and disclosure behavior is not fixed, but may be jointly moderated by individuals’ affective experiences and cognitive attitudes. Studies have provided evidence that negative experiences on social media, such as data theft, interpersonal conflicts, and cyberbullying, not only directly drive users to adopt privacy protection behaviors like identity masking and regulated use, but also indirectly influence such behaviors by activating privacy concerns and information disclosure awareness (Niu et al., 2024; Niu et al., 2025). Furthermore, Mazhar et al. (2025), drawing on the privacy paradox perspective, found that while awareness of online identity disclosure promotes privacy protection behaviors among social media users, this positive effect is negatively moderated by online privacy concerns—that is, as the level of concern increases, the efficiency with which awareness translates into action significantly diminishes. Together, these findings suggest that the relationship between risk perception and disclosure behavior is not simply linear.

In summary, although previous studies have revealed a relationship between privacy risk perception and privacy disclosure behavior, the findings have been inconsistent. The strength and direction of this relationship may be moderated by other psychological factors or individual characteristics. Consequently, the current investigation focuses on college students. By controlling for demographic variables such as gender, grade, and only-child status, we will further examine the relationship between privacy risk perception and privacy disclosure behavior. Additionally, We will explore potential mediating mechanisms and moderating variables to help elucidate the pathways and boundary conditions of this relationship.

### The mediating role of online social anxiety

Online social anxiety refers to the negative emotional experiences, such as tension and worry, that individuals experience during social interactions on social media platforms (Chen et al., 2023). It is often characterized by excessive concern about others’ evaluations after sharing content, persistent unease about the protection of personal privacy on social media, and fear of not receiving positive responses or facing social rejection when interacting with others (Hong et al., 2015). Communication Privacy Management Theory (CPM) emphasizes that individuals control the flow of personal information by establishing privacy boundaries (Petronio, 2002). When users perceive a weakened ability to control these boundaries or an increased risk of boundary violation, they experience strong psychological discomfort. In the social media environment, this discomfort often manifests as online social anxiety, a state of tension caused by the fear of losing control over privacy boundaries (Jiang et al., 2008).

Previous studies have documented that a higher level of privacy risk perception leads to higher levels of online social anxiety. The persistent visibility of social media means that users’ personal profiles may be accessed without authorization by others as they engage in online activities, and such personal information may be obtained without the user’s knowledge (Davidson and Farquhar, 2014). In this environment, users often lack mutually agreed-upon privacy rules with their numerous online contacts. This boundary ambiguity may lead to improper dissemination of information, which in turn negatively affects real-life interpersonal relationships (Niu and Meng, 2019). This psychological burden may directly trigger and exacerbate feelings of tension, worry, and self-censorship during online interactions, thereby resulting in higher levels of social anxiety.

Additionally, some studies have revealed that social anxiety significantly inhibits privacy disclosure behavior. Individuals with high social anxiety tend to reduce self-disclosure and other social activities due to excessive concerns about negative evaluations and privacy breaches. This hinders the establishment of good interpersonal relationships, leads to unmet social needs, and creates a vicious cycle (Liu et al., 2018). Research has confirmed that such individuals are often more restrained and inhibited in interpersonal interactions. Although they desire to make a good impression on others, they lack the confidence to self-disclosure and the necessary social skills, resulting in more restricted disclosure behaviors, including the disclosure of private information (Brand et al., 2016).

In summary, we hypothesized that privacy risk perception may affect college students’ self-disclosure behavior through online social anxiety. That is, online social anxiety may mediate the relationship between privacy risk perception and privacy disclosure behavior.

### The moderating role of privacy cynicism

Privacy cynicism refers to an individual’s negative and distrustful attitude toward privacy protection on internet platforms (Hoffmann et al., 2024). As a result, privacy protection is subjectively perceived as futile. Research has suggested that the cognitive tendency of cynicism profoundly shapes individual decision-making processes (Brockway et al., 2002). In the context of data surveillance, users with high privacy cynicism believe that privacy breaches is inevitable and that privacy protection is meaningless. This may lead them to abandon the protection of their personal information and to disclose more data online (Li J. H. et al., 2023). Van Ooijen et al. (2024) found that, for individuals with a high level of privacy cynicism, the negative relationship between perceived benefits and privacy protection behavior becomes a positive relationship. When individuals are highly skeptical about privacy protection, they may, based on cost-minimization logic, take protective measures when they perceive clear benefits. This suggests a behavioral pattern that is qualitatively different from the traditional rational decision-making model.

Based on relevant research, privacy cynicism may play an important moderating role between privacy risk perception and privacy disclosure behavior. Research has provided suggestive evidence that the level of social media fatigue among users is significantly positively associated with their personal information disclosure behavior. Since cynicism is a core manifestation of fatigue, the negative attitude held by users with high privacy cynicism may become a potential driving factor for them to disclose more personal information on the internet (Anderson and Agarwal, 2010; Cramer et al., 2016).

Privacy cynicism may also play an important role in the process through which privacy risk perception affects online social anxiety. Individuals with high cynicism have high psychological expectations regarding potential privacy risks. Even when they perceive significant privacy risks, these perceptions are less likely to translate into strong anxiety (Ye, 2025). This cognitive style of anticipated realization helps them remain relatively calm when facing potential privacy threats and avoid falling into excessive anxiety.

Privacy cynicism may also moderate the strength and direction of the effect of online social anxiety on privacy disclosure behavior. For individuals with high privacy cynicism, the inhibitory effect of online social anxiety on information disclosure is significantly weakened (Xie et al., 2018). Although these users experience social anxiety, they are convinced that privacy will not be protected and that any privacy protection measures are futile. As a result, they may adopt a more permissive information disclosure strategy. This cognitive style weakens the traditional association between risk perception and behavioral inhibition, so that anxiety no longer necessarily leads to a reduction in disclosure behavior.

**Figure 1:**
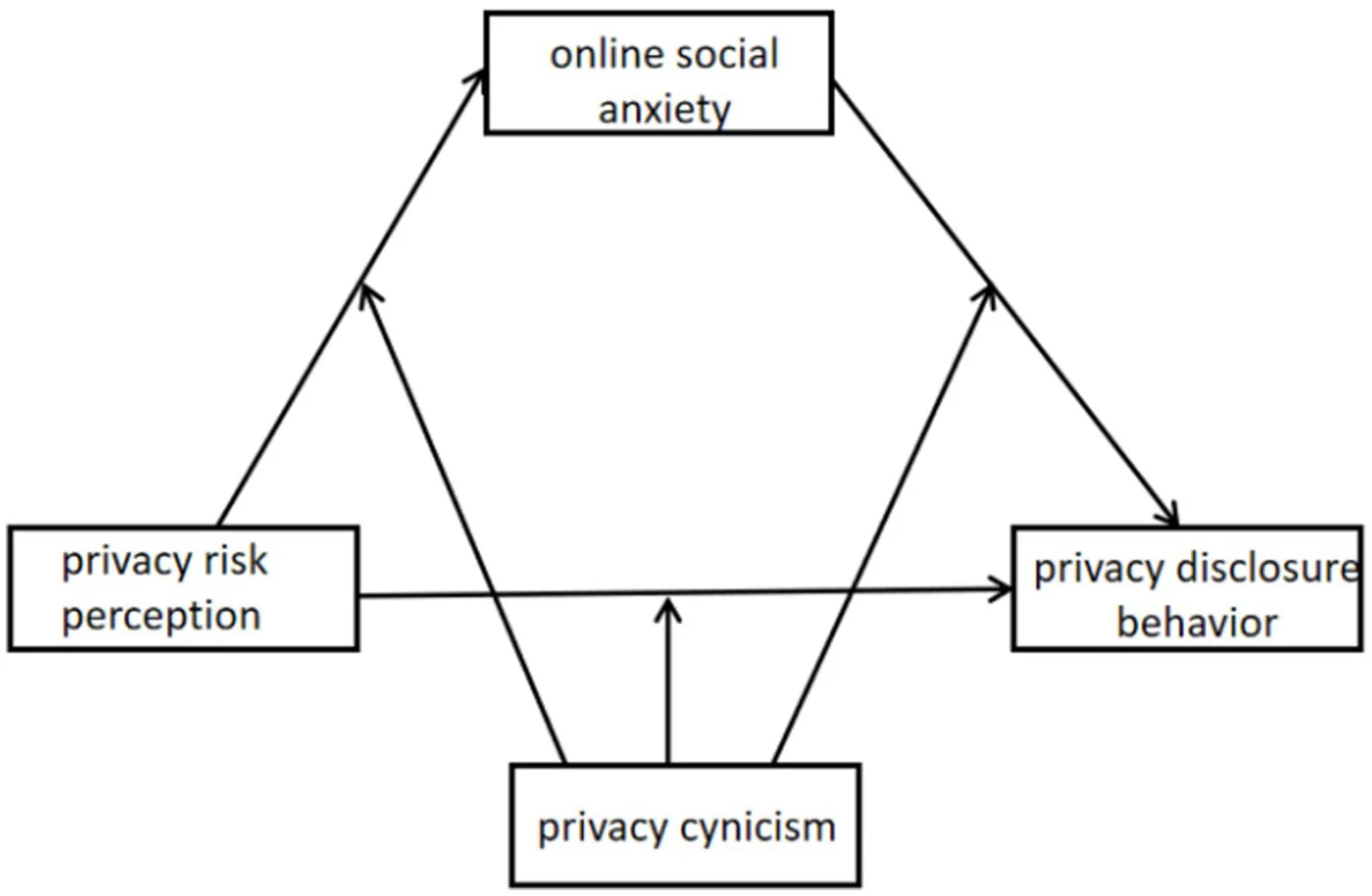
Research model.

Based on the above analysis, the present research investigates the factors influencing and mechanisms underlying college students’ privacy disclosure behavior. It will examine the direct effect of privacy risk perception, the mediating role of online social anxiety, and the moderating role of privacy cynicism. Regarding the relationship between privacy risk perception and privacy disclosure behavior, existing findings remain controversial. Some studies support a negative prediction, while others have reported no significant association. This discrepancy may stem from the exclusion of emotional and cognitive moderating factors. Nevertheless, according to PMT, risk perception, as a core cognitive variable in threat appraisal, should promote protective behaviors, that is, inhibit privacy disclosure behavior. Therefore, we propose the following hypotheses: (1) Privacy risk perception negatively predicts college students’ privacy disclosure behavior; (2) Online social anxiety mediates the relationship between privacy risk perception and privacy disclosure behavior; (3) The effect of privacy risk perception on privacy disclosure behavior, as well as the mediating role of online social anxiety in this relationship, are both moderated by privacy cynicism. The hypothesized model is illustrated in Figure 1.

## Methods

### Participants

The sample consisted of college student participants. A convenience sampling method was used. Participants were recruited from six universities in the Beijing-Tianjin-Hebei region. They voluntarily completed the questionnaires and signed informed consent forms. Inclusion criteria were: (1) informed consent from the participant; (2) normal cognitive ability and clear consciousness. Exclusion criteria were: (1) involuntary participation; (2) incomplete responses, patterned selections, or excessively long or short response times. A total of 1,293 anonymous questionnaires were collected. Among them, 1,276 were valid, yielding an effective response rate of 98.7%. Among the valid questionnaires, there were 488 male participants (38.2%) and 788 female participants (61.8%). Regarding grade level, 464 were freshmen (36.4%), 314 were sophomores (24.6%), 394 were juniors (30.9%), and 104 were seniors (8.1%). In terms of academic discipline, 541 majored in science and engineering (42.4%), 342 in social sciences (26.8%), and 393 in medicine (30.8%). There were 280 only-child participants (21.9%) and 996 non-only-child participants (78.1%). Regarding origin, 370 were from urban areas (29.0%) and 906 were from rural areas (71.0%). This study was approved by the Ethics Committee of North China University of Science and Technology (Approval No. 2025160).

### Measures

#### Perceived privacy risk measurement scale

This scale was developed by Dinev and Hart (2006). It is used to measure an individual’s perceived level of the unpredictability of personal information breaches and the consequences of such breaches. The scale is a single-dimensional self-report measure. It consists of four items, rated on a 5-point Likert scale (1 = strongly disagree, 5 = strongly agree). Higher scores indicate a stronger level of perceived privacy risk among participants. In the current sample, the reliability of the scale was Cronbach’s *α* alpha = 0.94.

#### Privacy disclosure behavior scale

This scale was developed by Dienlin and Trepte (2015). It is used to assess the actual extent to which individuals provide personal information to social media. The scale is a single-dimensional self-report measure. It consists of five items, rated on a 5-point Likert scale (1 = strongly disagree, 5 = strongly agree). Higher scores indicate a higher level of privacy disclosure behavior among participants. In the current sample, the reliability of the scale was Cronbach’s *α* alpha = 0.76.

#### Social anxiety scale for social media users

This scale was revised by Chen et al. (2020). It is used to assess the level of online social anxiety among individuals. The scale consists of three dimensions: evaluation anxiety, interaction anxiety, and privacy concerns. It includes a total of 20 items, rated on a 5-point Likert scale (1 = completely disagree, 5 = completely agree). Higher scores indicate a higher level of online social anxiety among participants. In the current sample, the Cronbach’s *α* coefficient of the total scale was 0.96. The Cronbach’s α coefficients for the three subscales were 0.93, 0.91, and 0.81, respectively.

#### Privacy cynicism scale

This scale was revised by Li J. H. et al. (2023). It is used to measure users’ levels in four aspects: distrust of social media, uncertainty about the use of personal information, powerlessness in privacy protection, and compliance in privacy disclosure. The scale consists of four dimensions (distrust, uncertainty, powerlessness, and compliance) and includes a total of 8 items. Items are rated on a 5-point Likert scale (1 = strongly disagree, 5 = strongly agree). Higher scores indicate a higher level of privacy cynicism among participants. In the current sample, the Cronbach’s α coefficient of the total scale was 0.86. The Cronbach’s α coefficients for the four subscales were 0.86, 0.81, 0.79, and 0.69, respectively.

#### Delineation of core constructs by psychological levels

To ensure conceptual clarity for subsequent statistical tests and the interpretation of effects, we first delineate the psychological levels of the four core constructs. Specifically, privacy risk perception falls under the cognitive appraisal level and reflects individuals’ subjective judgments of the likelihood and severity of information breaches. Online social anxiety belongs to the emotional experience level and reflects the tension and unease individuals feel during social media interactions. Its “privacy concern” dimension reflects emotional worry, rather than cognitive risk assessment. Privacy cynicism belongs to the attitudinal belief level and reflects individuals’ overall negative beliefs about the effectiveness of privacy protection. Privacy disclosure behavior belongs to the behavioral response level and reflects actual self-disclosure actions.

### Data analysis

Descriptive statistics, reliability analysis, and Pearson correlation analysis were performed using SPSS 24.0. The Harman single-factor test was conducted to test for common method bias. Principal component factor analysis was performed on all items. The analysis extracted five factors with eigenvalues greater than 1. The variance explained by the first factor was 36.69%, which is below the critical value of 40%. This indicates that common method bias is not a serious concern in the current data. Previous studies have documented that gender is significantly associated with privacy concerns, with females generally exhibiting higher privacy awareness than males (Baruh et al., 2017). This may stem from the influence of gender socialization on threat appraisal processes. Grade level is related to college students’ self-disclosure on social networking sites, with lower-grade students generally displaying more disclosure behaviors (Lin et al., 2023). This may be because lower-grade students face a new social environment and urgently need to establish interpersonal relationships and seek social recognition through self-disclosure, thus showing greater initiative in disclosing information on social media. Academic major influences individuals’ attitudes and habits toward information sharing, and students from different majors differ in their degree of self-disclosure (Lin et al., 2023). Disciplinary training may cultivate distinct analytical thinking styles and digital literacy, which in turn affect individuals’ evaluation of privacy threats and disclosure benefits. Place of origin reflects, to some extent, differences in college students’ level of digital exposure (Zeng, 2011). Early digital exposure may influence individuals’ privacy awareness and their judgment and handling of privacy issues. If this factor is not controlled for, it may confound the accurate estimation of the relationships among risk perception, anxiety, and behavior in our analysis. Whether one is an only child is associated with family resources and social support networks, which indirectly affect individuals’ social anxiety and disclosure behaviors (Liao and Shi, 2016). Family structure differences may shape different patterns of interpersonal dependence and emotion regulation strategies, which are further linked to online social anxiety and self-disclosure tendencies. Therefore, to exclude the potential confounding effects of these demographic variables, we included gender, grade, major, place of origin, and only-child status as control variables. AMOS 24.0 was applied to conduct confirmatory factor analysis and path analysis for the mediation effect. The PROCESS-Macro v3.3 was adopted to test the moderated mediation effect, employing bias-corrected Bootstrap method with 5,000 resamples. The confidence level for the confidence interval was 95%. A *p*-value of less than 0.05 is considered statistically significant.

## Results

### Descriptive statistics and correlation analysis

The descriptive statistics for all core variables were: privacy risk perception (15.30 ± 4.19), privacy disclosure behavior (12.50 ± 3.33), online social anxiety (58.52 ± 14.13), and privacy cynicism (24.77 ± 5.33). A Pearson correlation analysis was conducted on the four core variables. The results showed that pairwise correlations among privacy risk perception, privacy disclosure behavior, online social anxiety and its dimensions, and privacy cynicism were all significantly positive (*p* < 0.01) (Table 1).

**Table 1:** Descriptive statistics and correlation analysis results (*n* = 1,276).

Variables	*M*	*SD*	1	2	3	4	5	6
1. Privacy risk perception	15.30	4.19						
2. Privacy disclosure behavior	12.50	3.33	0.39**					
3. Online social anxiety	58.52	14.13	0.28**	0.24**				
4. Evaluation anxiety	29.42	7.36	0.27**	0.26**	0.97**			
5. Interaction anxiety	16.86	4.60	0.17**	0.19**	0.93**	0.84**		
6. Privacy concerns	12.24	3.06	0.37**	0.21**	0.89**	0.80**	0.77**	
7. Privacy cynicism	24.77	5.33	0.38**	0.31**	0.55**	0.55**	0.45**	0.53**

*p < 0.05, **p < 0.01.

### Measurement model evaluation

Before testing the research hypotheses, this study used AMOS 24.0 to conduct a confirmatory factor analysis (CFA) on the four core constructs to evaluate the fit of the measurement model. The results showed: χ^2^/df = 6.20, CFI = 0.90, TLI = 0.89, RMSEA = 0.06, SRMR = 0.07. The χ^2^/df ratio slightly exceeded the recommended threshold of 5, which may be attributable to the relatively large sample size and the substantial number of measured items in the current dataset (the χ^2^ statistic is known to be sensitive to both sample size and the number of indicators). Given that the CFI reached the acceptable cutoff of 0.90, and both RMSEA and SRMR were below the recommended criterion of 0.08, the measurement model was judged to have an acceptable level of fit, allowing further analyses to proceed. Meanwhile, the standardized factor loadings of all measurement items ranged from 0.40 to 0.95, and all were statistically significant (*p* < 0.001). No items were deleted due to low factor loadings. Furthermore, the composite reliability (CR) values for all latent variables exceeded the acceptable threshold of 0.70. The average variance extracted (AVE) values ranged from 0.39 to 0.81, indicating basically acceptable convergent validity. It is worth noting that the Cronbach’s *α* coefficient for the “compliance” sub-dimension of privacy cynicism was 0.69, slightly below the conventional cutoff of 0.70. This may be because this sub-dimension consists of only three items, as shorter scales tend to produce lower α values. However, given that the CR value for this variable still exceeded 0.70, its internal consistency remains within an acceptable range for practical research purposes.

Overall, the measurement model exhibited acceptable psychometric properties, confirming that the data are suitable for further substantive analysis and hypothesis testing (Table 2).

**Table 2:** Confirmatory factor analysis fit indices.

Latent variable	CR	AVE	Range of standardized factor loadings
Privacy risk perception	0.95	0.81	0.83–0.95
Privacy disclosure behavior	0.75	0.39	0.40–0.75
Online social anxiety	0.96	0.54	0.59–0.82
Privacy cynicism	0.86	0.44	0.44–0.85

### Mediating effects of online social anxiety

**Figure 2:**
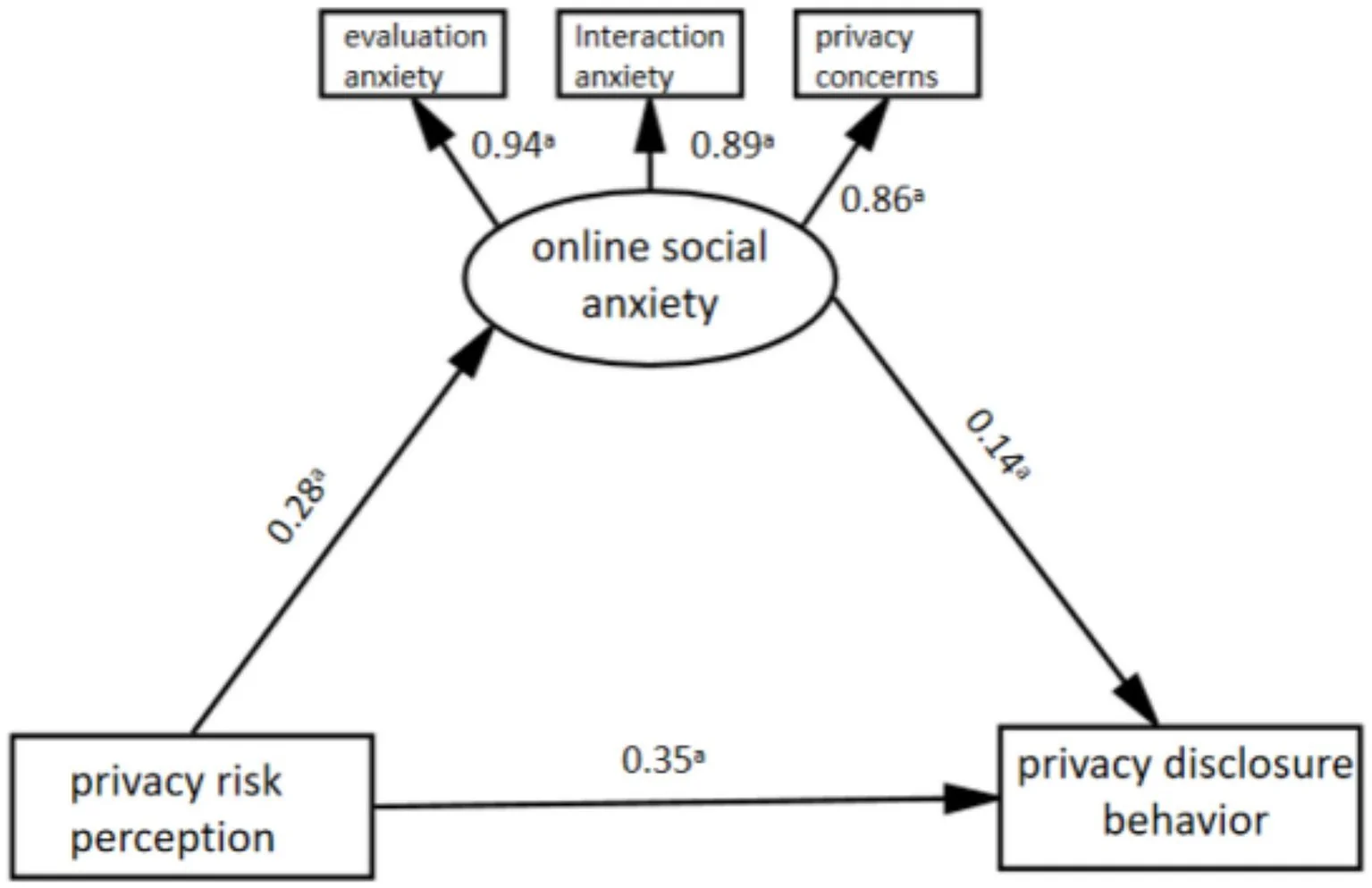
Mediation model of online social anxiety on the relationship between privacy risk perception and privacy disclosure behavior.

After standardizing the data, a model was established in AMOS 24.0 to test the mediation effect. The path analysis showed that privacy risk perception was significantly and positively associated with privacy disclosure behavior (*β* = 0.35, *p* < 0.001). Privacy risk perception was also significantly and positively associated with online social anxiety (*β* = 0.28, *p* < 0.001). Online social anxiety was significantly and positively associated with privacy disclosure behavior(*β* = 0.14, *p* < 0.001). The bias-corrected percentile Bootstrap method indicated that the mediating effect of online social anxiety between privacy risk perception and privacy disclosure behavior was significant, with an effect value of 0.04 (95%CI = [0.02, 0.07]). The mediating effect accounted for 10.26% of the total effect (Table 3 and Figure 2).

**Table 3:** Decomposition of total effect, direct effect, and mediating effect.

Item	Effect value	Standard error	95%CI	Effect size(%)
Total effect	0.39	0.03	0.33 ~ 0.45	
Direct effect	0.35	0.03	0.28 ~ 0.40	89.74
Indirect effect	0.04	0.01	0.02 ~ 0.07	10.26

The above mediation analysis results suggest a significant positive correlation between privacy risk perception and privacy disclosure behavior. This finding is inconsistent with research hypothesis H1; therefore, H1 is not supported. This positive association appears to corroborate the privacy paradox phenomenon. Among college students, high levels of risk perception do not inhibit behavior as traditional threat assessment theories would predict. This preliminarily suggests that their decision-making process may be more consistent with the logic of “benefit–risk trade-off” in privacy calculus theory. Furthermore, the indirect effect of online social anxiety between the two variables is significant, suggesting that emotional experience may serve as an important bridge connecting cognitive appraisal and behavioral response. Research hypothesis H2 is supported.

### The moderating effect of privacy cynicism

Using Model 59 in the PROCESS macro, the moderating role of privacy cynicism was tested while controlling for gender, grade, major, place of origin, and only-child status. This model also includes the main effect of privacy cynicism and two product interaction terms. These terms involve privacy cynicism with privacy risk perception and with online social anxiety, respectively. The model specification differs from that of the simple mediation model in Table 2. Therefore, the direct effect coefficient of privacy risk perception on privacy disclosure behavior shows a numerical difference. However, the two sets of results are not conflicting. The regression output showed that after including privacy cynicism in the model, privacy cynicism was significantly and positively associated with privacy disclosure behavior (*β* = 0.14, *SE* = 0.03, *p <* 0.001). The interaction term of privacy risk perception and privacy cynicism was significantly associated with privacy disclosure behavior (*β* = −0.05, *SE* = 0.02, *p* < 0.05). Privacy cynicism was significantly and positively associated with online social anxiety(*β* = 0.50, *SE* = 0.03, *p* < 0.001). The interaction term of privacy risk perception and privacy cynicism was significantly associated with online social anxiety (*β* = −0.10, *SE* = 0.02, *p* < 0.001). The interaction term of online social anxiety and privacy cynicism was not significantly associated with privacy disclosure behavior (*β* = −0.03, *SE* = 0.02, *p >* 0.05). These findings together suggest that privacy cynicism moderates the relationships between privacy risk perception and privacy disclosure behavior, as well as between privacy risk perception and online social anxiety (Table 4).

**Table 4:** Test of moderated mediation.

Predictor	Online social anxiety			Privacy disclosure behavior		
	*β*	*SE*	*t*	*β*	*SE*	*t*
Gender	0.05	0.05	0.99	0.01	0.05	0.01
Grade	0.01	0.02	0.49	0.04	0.03	1.67
Major	0.02	0.02	0.67	−0.02	0.03	−0.90
Place of origin	0.03	0.05	0.58	0.03	0.06	0.42
Only-child status	0.02	0.06	0.31	−0.03	0.06	−0.42
Privacy risk perception	0.04	0.03	1.46	0.29	0.03	10.14***
Online social anxiety				0.07	0.03	2.06*
Privacy cynicism	0.50	0.03	19.70***	0.14	0.03	3.92***
Privacy risk perception × Privacy cynicism	−0.10	0.02	−5.37***	−0.05	0.02	−2.21*
Online social anxiety × Privacy cynicism				−0.03	0.02	−1.44
*R* ^2^			0.33			0.20
*F*			76.31***			30.71***

*p < 0.05, **p < 0.01, ***p < 0.001.

**Figure 3:**
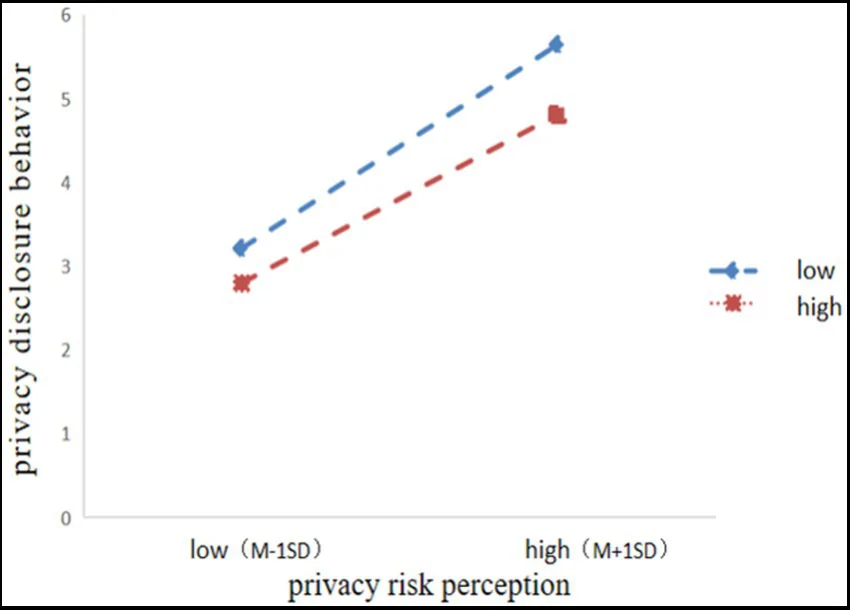
The moderating role of privacy cynicism on the relationship between privacy risk perception and privacy disclosure behavior.

**Figure 4:**
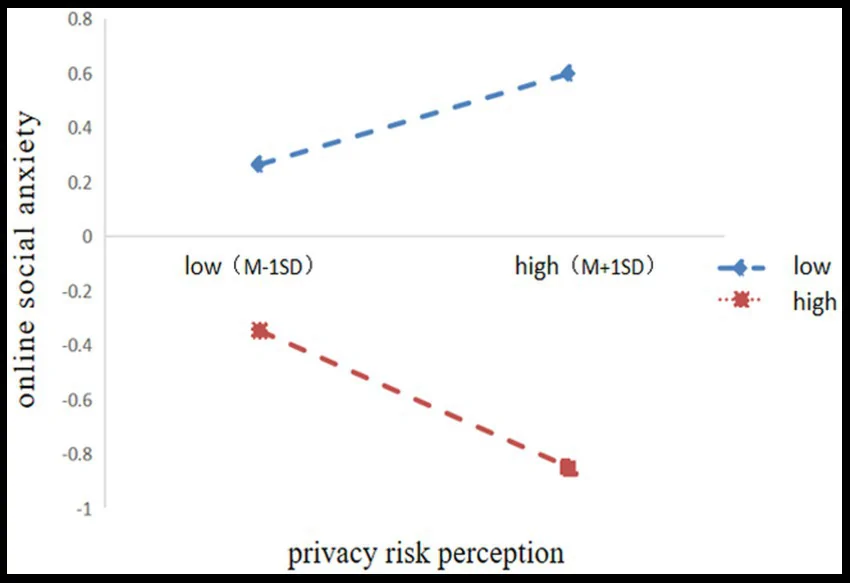
The moderating role of privacy cynicism on the relationship between privacy risk perception and online social anxiety.

Finally, privacy cynicism was divided into high and low groups based on one standard deviation above and below the mean. Simple slope plots were drawn to further analyze the moderating effect of privacy cynicism. The analysis (Figure 3) showed that for college students with low privacy cynicism, privacy risk perception was significantly and positively associated with privacy disclosure behavior (*B*_simple_ = 0.34, *t* = 10.72, *p* < 0.001). For college students with high privacy cynicism, the association between privacy risk perception and privacy disclosure behavior was weaker (*B*_simple_ = 0.24, *t* = 6.26, *p* < 0.001). Next, the moderating role of privacy cynicism on the relationship between privacy risk perception and online social anxiety was analyzed. The results (Figure 4) showed that in the low privacy cynicism group, privacy risk perception was significantly and positively associated with online social anxiety (*B*_simple_ = 0.12, *t* = 4.68, *p* < 0.001). In the high privacy cynicism group, the association between privacy risk perception and online social anxiety was not significant (*B*_simple_ = −0.05, *t* = −1.44, *p* > 0.05).

These results indicate that privacy cynicism plays a significant moderating role. In the relationship between privacy risk perception and privacy disclosure behavior, privacy cynicism significantly weakened the strength of their association. Regarding the relationship between privacy risk perception and online social anxiety, privacy risk perception was significantly and positively associated with online social anxiety in the low privacy cynicism group, but this association was not significant in the high privacy cynicism group. Both the direct effect of privacy risk perception on privacy disclosure behavior and the mediating effect of online social anxiety showed a decreasing trend (Table 5).

**Table 5:** Analysis of the moderating role of privacy cynicism.

Path	Privacy cynicism	Effect value	Boot SE	Boot LLCI	Boot ULCI
Direct effect	M-SD	0.34	0.03	0.28	0.40
	M	0.29	0.03	0.24	0.35
	M + SD	0.24	0.04	0.17	0.32
Mediating effect of online social anxiety	M-SD	0.12	0.03	0.07	0.17
	M	0.04	0.03	−0.01	0.09
	M + SD	−0.05	0.03	−0.11	0.02

Based on the above results, research hypothesis H3 is partially supported. Specifically, privacy cynicism has significant moderating effects on the direct path and on the first stage of the mediation path. However, its moderating effect on the second stage of the mediation path is not significant. This suggests that the moderating function of privacy cynicism may be path-specific. It appears to operate mainly at the cognitive transformation stage between risk perception and affective responses, and at the stage of the direct effect of cognition on behavioral decision-making. Yet it does not significantly moderate the intensity with which anxious emotions translate into actual behavior.

## Discussion

The present research combined an emotional response (online social anxiety) with a cognitive attitude (privacy cynicism) to further clarify the complex relationship between privacy risk perception and privacy disclosure behavior. Based on the Protection Motivation Theory, the Communication Privacy Management Theory, and the Privacy Calculus Theory, a moderated mediation model was constructed. This model may deepen the understanding of the internal mechanism through which privacy risk perception influences disclosure behavior.

### The effect of privacy risk perception on college students’ privacy disclosure behavior

The empirical results suggest that college students’ privacy risk perception is significantly and positively associated with their privacy disclosure behavior. That is, when college students perceive higher privacy risks in social media use, they still tend to disclose more personal information. This observation suggests that the linear logic linking risk perception to protective behavior, as assumed by PMT, is not supported in the social media context of college students.

This finding differs from those of previous studies (Peng et al., 2022; Niu and Meng, 2019), yet seems broadly consistent with the PCT. This theory suggests that when perceived situational benefits are sufficiently salient, individuals may voluntarily disclose information for short-term gains, even when they are aware of potential privacy risks (Culnan and Armstrong, 1999; Hallam and Zanella, 2017). A study by Hashmat and Azeema (2025) reported a significant negative association between age and self-disclosure. Specifically, younger users aged 18 to 25 tend to show lower privacy concerns when disclosing personal information compared to older users. Contemporary college students, who are immersed in the instant gratification brought by the Internet, tend to associate privacy disclosure with multiple benefits (Lan, 2017).

From the perspective of social benefits, self-disclosure helps build and maintain interpersonal relationships. It also helps gain peer attention and social recognition. For college students, who are in a critical period of self-identity and social capital accumulation, the sense of belonging provided by online platforms may be a key factor in encouraging the disclosure of personal information. From the perspective of utilitarian benefits, social media platforms use material incentives and personalized recommendations to encourage users to disclose data. Users tend to view information disclosure as a necessary condition for accessing free resources and convenient services (Li, 2023). Therefore, although college students appear to recognize privacy risks, they may still choose to disclose information under the influence of multiple benefits such as social recognition, practical value, and self-presentation, showing a benefit-prioritizing decision tendency.

Meanwhile, under the current online environment, it has become common for social media apps to obtain users’ private information (Wu and Yao, 2022). Frequent users of social media, or “heavy users,” often experience increased risk perception during their frequent interactions. However, this heightened awareness appears to coexist with, rather than curtail, their continued enjoyment of social networks, Whose privacy disclosure behavior seems to continue increasing (Kwon and Johnson, 2015). This pattern collectively suggests that, in the current social media ecology, risk perception and disclosure behavior may co-exist rather than operate in opposition.

### Mediating effects of online social anxiety

Having established the direct association, we next examined its underlying mechanism. The mediation analysis found that online social anxiety may play a partial mediating role between college students’ privacy risk perception and privacy disclosure behavior. Specifically, college students with higher privacy risk perception also experienced higher online social anxiety, a pattern consistent with previous research (Davidson and Farquhar, 2014). Concerns about privacy and others’ evaluations are important sources of user anxiety. College students perceive the possibility of information collection and data misuse in the social media environment as potential threats to their personal image, social relationships, and privacy space. They may also feel unable to fully control or avoid these risks. This cognitive appraisal pattern is often accompanied by negative emotions, such as anxiety and worry (Cong et al., 2025; Josol and Albon, 2025).

The findings also indicated that college students with higher online social anxiety tended to disclose more personal information. Previous studies have associated negative emotional states to specific social media behaviors (Lee, 2014). When individuals appraise privacy risks as social threats, they experience anxiety. To alleviate this discomfort, they may habitually turn to online platforms for emotional regulation. Disclosing personal information becomes one way to relieve negative emotions. In addition, the immediate benefits of reducing anxiety may outweigh privacy costs in their subjective trade-off. This pattern may explain why privacy disclosure increases in anxiety-provoking situations (Zhu et al., 2025; Wang et al., 2024).

### The moderating effect of privacy cynicism

With the mediating mechanism confirmed, we further probed the boundary conditions of this process. The moderated mediation analysis suggested that the moderating role of privacy cynicism was limited to the direct path and the first stage of the mediation path. Moreover, the moderated mediation effect was significant only under the condition of low privacy cynicism. This suggests that the moderating effect of privacy cynicism on the mediation process is relatively limited and conditional.

Regarding the direct path, as the level of privacy cynicism increased, the strength of the association between privacy risk perception and privacy disclosure behavior decreased significantly. This indicates that a cynical attitude may weaken the link between cognition and behavior. For users with high privacy cynicism, although they are aware of the potential risks of personal information being misused in the social media environment, their decision-making process appears to be often accompanied by a strong sense of powerlessness and distrust (Hoffmann et al., 2024). They believe that, under the current internet technology and online environment, efforts to protect privacy are futile. As a result, they develop a passive resignation. Their disclosure behavior is more related to habits, convenience, or instant gratification (Li J. H. et al., 2023). This significantly reduces the influence of privacy risk perception on their behavioral decisions. This finding underscores the complexity of users’ psychological processes in privacy decision-making. It suggests that when analyzing users’ privacy disclosure behavior, in addition to considering their level of risk perception, more attention might be paid to underlying attitudinal factors such as privacy cynicism. These factors may play a role in moderating the traditional relationship between risk and behavior.

Regarding the indirect path, the moderating role of privacy cynicism is only observed in the first stage. Individuals with low privacy cynicism are more sensitive to privacy issues. They tend to carefully evaluate and process privacy-related information (Hoffmann et al., 2024). Individuals who perceive privacy risks often experience anxiety due to concerns about their personal privacy and security (Caillan, 2026). In contrast, individuals with high privacy cynicism may pay less attention to the threatening nature of risks from the outset. When they enter social situations, their cynical mindset reduces the gap between actual risk and psychological expectation, thereby potentially exerting an adaptive regulatory effect on anxiety. As a result, individuals with high cynicism present a unique psychological profile when facing privacy risks: high risk perception, low anxiety experience, and high disclosure behavior. This provides a new perspective for explaining the privacy paradox. Some users are not unaware of the risks; rather, their cynical attitude helps them cognitively and emotionally adapt to the risky environment (Yu et al., 2025; Khan et al., 2023).

The moderating effect of privacy cynicism on the path from online social anxiety to privacy disclosure behavior was not significant. This might be because the motivation for emotional regulation in stressful situations plays a relatively more significant role in affecting behavior compared to rational cognitive deliberation. Individuals with high social anxiety may engage in strategic self-disclosure in the social media environment to compensate for their perceived insufficiency and uncertainty in self-presentation during real-time interactions. By shaping their online image to gain social approval, thereby potentially alleviating anxiety (Kay and Eibach, 2013). According to the affect-cognition framework, under high emotional arousal, the affective system often takes precedence over the cognitive reflective system (Loewenstein et al., 2001). Therefore, regardless of whether individuals generally hold a cynical attitude toward privacy, the immediate emotional motivation to disclose in order to relieve anxiety in specific social situations may be stronger. This may mask the moderating effect of privacy cynicism.

### Implications

#### Theoretical implications

The present framework is based on Protection Motivation Theory, Communication Privacy Management Theory, and Privacy Calculus Theory. It constructs and tests a moderated mediation model. The model examines the roles of online social anxiety and privacy cynicism in the relationship between college students’ privacy risk perception and privacy disclosure behavior.

First, this work provides additional empirical evidence for the privacy paradox. Previous research has often focused on the negative association between privacy risk perception and disclosure behavior. In contrast, our findings point to a positive association between privacy risk perception and privacy disclosure behavior among college students. This finding is consistent with the benefit-first decision logic proposed by PCT. It suggests that even when individuals are clearly aware of risks, their disclosure behavior may still be associated with short-term benefits such as social recognition and instant gratification. This finding offers a complementary perspective for understanding the privacy behavior of digital-native college students.

Second, we incorporated online social anxiety as a mediating variable into the privacy decision-making model. The analysis showed that privacy risk perception was positively associated with online social anxiety. Online social anxiety was positively associated with privacy disclosure behavior. Moreover, online social anxiety had a significant mediating effect on the relationship between privacy risk perception and privacy disclosure behavior. This finding helps clarify the mediating role of online social anxiety between cognitive appraisal and behavioral response. These results suggest that negative emotions may have a dual role between risk perception and behavior. It may enrich the understanding of the emotional mediation between threat appraisal and coping behavior within PMT.

Third, this study examined the dual moderating role of privacy cynicism. Privacy cynicism significantly weakened the strength of the associations between privacy risk perception and privacy disclosure behavior, as well as between privacy risk perception and online social anxiety. This indicates that a cynical mindset may reshape the traditional relationships among risk, anxiety, and behavior. This finding provides additional insights into the concept of boundary control in CPM.

#### Practical implications

The findings of this study have practical value for cybersecurity education, mental health intervention, and social media platform design among college students.

In terms of cybersecurity education, simply strengthening risk perception may not reduce privacy disclosure behavior. Education might consider shifting from risk warning to need guidance, it is essential to understand the deep motivations behind college students’ disclosure behavior, such as social recognition, sense of belonging, and instant gratification. Through offline club activities, positive online community feedback, and rational decision-making training, we can help students gain psychological satisfaction without relying on excessive disclosure. At the same time, we should guide them to recognize the immediate discount bias between benefits and risks. This helps improve their ability to think about long-term consequences.

Regarding online social anxiety, our findings indicate that anxiety plays a driving role in the relationship between risk perception and disclosure behavior. Some college students use privacy disclosure as an emotion regulation strategy. Mental health practitioners should treat online social anxiety as an early warning indicator of privacy risk. Through emotion regulation training, such as mindfulness and cognitive reappraisal, they can help students develop healthier coping styles. This breaks the vicious cycle where anxiety drives disclosure and disclosure brings secondary risks.

For social media platforms, individuals with high privacy cynicism give up self-restraint. Platforms should rebuild user trust through transparent data policies, easy-to-use privacy settings, and timely breach response mechanisms. They should avoid designing forced disclosure interfaces.

Universities should establish an integrated education system that combines cybersecurity and mental health. They should include privacy protection, anxiety management, and media literacy in compulsory courses. They can design scenario-based simulation cases to help students experience the decision-making process from risk perception to emotional response to behavioral choice. This improves their metacognitive monitoring ability. In addition, for students who have experienced information breach or fraud, universities should provide psychological counseling and legal support. This prevents them from falling into deeper cynicism and excessive disclosure.

### Limitations and future research directions

Several limitations of this work should be noted. First, this study adopted a cross-sectional design, which precludes the determination of causal relationships among the variables. Future research could adopt longitudinal designs to collect data across multiple time points, thereby more clearly revealing potential causal pathways. Second, the sample exhibited some demographic imbalances, particularly in gender distribution and place of origin, which may limit the generalizability of the findings to the broader population of Chinese college students. Future research should improve sampling strategies and expand the coverage of institution types and geographical regions to enhance external validity. Third, although the core constructs investigated here belong to four distinct psychological levels, namely cognition, emotion, attitude, and behavior, the scale items are semantically related as they all pertain to privacy issues. Future research could employ experimental paradigms or experience sampling methods, using situational manipulation and real-time tracking, to more adequately disentangle the independent effects of each psychological level. Furthermore, while the present work provides empirical evidence for the privacy paradox phenomenon, the empirical model did not directly measure perceived benefits. Consequently, the discussion regarding the “benefit-prioritizing” decision tendency remains a theoretical interpretation inferred from existing literature rather than a directly tested effect. Future research could incorporate both perceived risk and multidimensional perceived benefits into the model, and examine their interaction effects or construct a dual-path mediation model, to more comprehensively test the core assumptions of PCT. Additionally, some subscales in the current dataset exhibited relatively low reliability, which may increase measurement error and attenuate the precision of estimates for the moderating effect of privacy cynicism. Future research should revise item wording or add items to improve internal consistency, and could also refine the conceptualization of these dimensions and develop more robust measurement tools. Finally, this study relied on self-report data and used only Harman’s single-factor test to examine common method bias. Although the test result was within an acceptable range, this single post-hoc diagnostic approach has limited sensitivity. Future research could introduce marker variable techniques or control for an unmeasured latent method factor to more rigorously test and correct for common method bias, thereby strengthening the robustness of the conclusions.

## Conclusion

(1) Privacy risk perception is significantly and positively associated with privacy disclosure behavior. (2) Online social anxiety partially mediates the relationship between privacy risk perception and privacy disclosure behavior. (3) Privacy cynicism moderates both the relationship between privacy risk perception and privacy disclosure behavior, and the relationship between privacy risk perception and online social anxiety.

## Data Availability

The data analyzed in this study is subject to the following licenses/restrictions: Confidentiality of research. The raw data supporting the conclusions of this article will be made available by the authors, without undue reservation. Requests to access these datasets should be directed to Shuyan Huang 964261154@qq.com.
